# Turner et al. Reply to “Emergence of the Same Successful Clade among Distinct Populations of *emm*89 *Streptococcus pyogenes* in Multiple Geographic Regions”

**DOI:** 10.1128/mBio.01883-15

**Published:** 2015-12-01

**Authors:** Claire E. Turner, Theresa Lamagni, Matthew T. G. Holden, Sophia David, Michael D. Jones, Laurence Game, Androulla Efstratiou, Shiranee Sriskandan

**Affiliations:** a Infectious Diseases & Immunity, Department of Medicine, Imperial College London, London, United Kingdom; b Public Health England, London, United Kingdom; c Pathogen Genomics, The Wellcome Trust Sanger Institute, Cambridge, United Kingdom; d School of Medicine, University of St. Andrews, St. Andrews, United Kingdom; e MRC Clinical Sciences Centre, Hammersmith Hospital, London, United Kingdom

## REPLY

We are pleased that Friães et al. have elected to examine *emm*89 strains in Portugal (1); it was our hope that other groups would evaluate *emm*89 strains from elsewhere, using either whole-genome sequencing (WGS) or the PCR method that we described in our report (2). Subsequent to our publication (2), Zhu et al. used WGS to identify variants of the *nga*-*slo* promoter among *emm*89 isolates in the United States, Finland, and Iceland (3) indicative of the new emergent *emm*89 clade in these countries. Although they did not use WGS, Friães et al. demonstrated that it is likely that the emergent *emm*89 clade that we identified in the United Kingdom and elsewhere is also present in Portugal (1).

Friães and colleagues have noted that the focus on different aspects of *emm*89 strains in the various studies (2–4) makes it hard to determine if the same clade had disseminated in different regions. In our original report, we aimed to provide a single comprehensive description of all six regions of recombination that characterize this emergent *emm*89 clade (2). Two of these regions, the *nga*-*slo* locus (region 2) and the *hasABC* capsule locus (region 6), were highlighted because of their phenotypic significance; it was beyond the scope of our paper to undertake in-depth analysis of all of the regions. We did, however, clearly demonstrate enhanced NGA-SLO toxin expression and stated that this could be due to a single polymorphism or several polymorphisms within the *nga*-*slo* locus and promoter or elsewhere in the genome. Indeed, it may be misleading to focus on a single region or multiple regions of recombination without understanding of the collective impact of all of the changes. In our sampling of the *emm*89 population, we failed to identify isolates with an intermediate number of recombination regions, raising the possibility that all six remodeled regions promoted the success of this lineage, not simply the two that have been highlighted.

**FIG 1:**
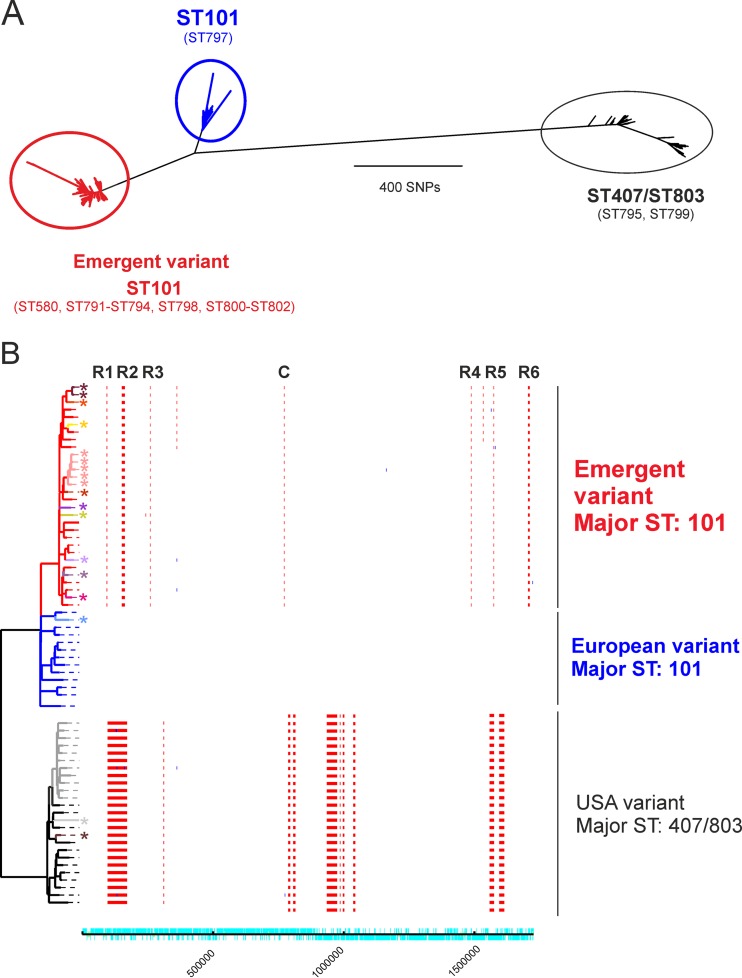
Phylogenetic analysis of *emm*89 population. (A) Three lineages were identified in the *emm*89 population based on the maximum likelihood phylogeny of SNPs identified after mapping to the H293 reference strain of the sequences of 868 *emm*89 strains from both the United Kingdom study (2) and the United States/Finland/Iceland study (3), where ST could be confidently assigned. We identified 16 sequence types (ST101, *n* = 621; ST407, *n* = 160; ST580, *n* = 3; ST791, *n* = 2; ST792 to ST800, *n* = 1 each; ST801, *n* = 20; ST802, *n* = 1; ST803, *n* = 52). Two lineages were equivalent to those in the United Kingdom study (2) which identified an emergent clade type (red) from a previous lineage (blue) where the major ST was ST101 with some single-locus variants (identified in parentheses). These lineages are also associated with the previously termed capsular *nga-slo* promoter “variant 2” strains (blue) and acapsular *nga-slo* promoter “variant 3” strains (red). A third lineage (black) was also identified, representing ST407 and ST803 and two other sequence type variants (ST795 and ST799), equivalent to the previously termed capsular *nga-slo* promoter “variant 1” strains. (B) Regions of recombination in a subset of 68 strains representing all of the sequence types identified compared to the reference strain H293 (ST101) were predicted using Gubbins (5). Branch colors represent lineages and sequence types. Emergent variant ST101 (red) and other single-locus variants (indicated with an asterisk [*]) are shown in the following order from the top of the tree: ST791 (maroon), ST802 (orange), ST796 (yellow), ST801 (pink), ST798 (dark red), ST792 (purple), ST580 (green), ST793 (lilac), ST794 (grey-purple), ST800 (bright pink). Previously dominant “European” variant ST101 (blue) and single-locus variant ST797 (pale blue) are shown, as are previously dominant “United States” variants ST407 (black) and ST803 (grey) and single-locus variants ST799 (pale grey) and ST795 (brown). Regions of recombination identified in each strain are shown as vertical red lines (indicating recombination on internal nodes) or blue lines (indicating recombination on terminal branches), and genome coordinates are given on the bottom line (turquoise). The six previously identified regions of recombination (R1 to R6) were present in the emergent clade strains of ST101 and other single-locus variants within this lineage, along with a variation in the clustered regularly interspaced short palindromic repeat (CRISPR) region (column C) (2). The United States variant ST407/ST803 lineage had several large regions of predicted recombination compared to the H293 reference strain, including one region which encompassed the MLST *yqil* locus and introduced the sequence type variation.

To more clearly illustrate the emergence of the new *emm*89 clade, we combined WGS data from isolates from the United States, Finland, and Iceland reported by Zhu et al. (3) for which we could confidently determine the sequence type (ST) (*n* = 737) with our data for United Kingdom and Swiss strains (2) (*n* = 131). We identified three distinct lineages (Fig. 1A) that are linked to the three previously identified *nga*-*slo* promoter variants (3). In agreement with Friães et al., the original United Kingdom *emm*89 population lineage (with *nga*-*slo* promoter “variant 2”) and the emergent acapsular *emm*89 clade (with *nga*-*slo* promoter “variant 3”) are both predominantly ST101; any ST variants within this clade are limited to single-locus variants. Isolates from Finland and Iceland, with a few minor exceptions, clustered either with the original United Kingdom *emm*89 ST101 lineage or with the new emergent ST101 clade described in our original paper (2). The majority of isolates from the United States, however, clustered either with the third identified lineage (with *nga*-*slo* promoter “variant 1”), dominated by ST407 and ST803, or with the new emergent ST101 clade. It appears that, as in our original findings in the United Kingdom, prior to 2007 to 2008, the dominant *emm*89 lineage in European countries was an ST101-like lineage but that the dominant lineage in the United States was an ST407/ST803-like lineage. Since 2007 to 2008, the new emergent variant, highly likely to have arisen within the ST101 background through recombination, has risen to dominance in both Europe and the United States. All members of this emergent clade have the same six regions of recombination, of which the *nga*-*slo* and *hasABC* regions are just two.

The ST407/ST803-like lineage sequenced by Zhu et al. (3) was infrequent in European countries and is separated from the two ST101-like lineages by a high level of core single nucleotide polymorphisms (SNPs) (Fig. 1A). In comparison to the *emm*89 H293 reference (ST101), this lineage contains its own distinct predicted pattern of recombination (Fig. 1B). Given the level of difference between ST101 and ST407/ST803, the success of the ST101 emergent clade in both North America and Europe may therefore reflect a more general competitive advantage of this new lineage. The findings also highlight a propensity of the genetic background of *emm*89 to undergo recombination.

We agree that the emergent clade has most likely acquired a competitive advantage through enhanced transmission and/or a propensity for superficial infections. In 2009, a year when the incidence of *emm*89 invasive infections peaked in the United Kingdom (2), *emm*89 accounted for 16% of all 135 noninvasive *Streptococcus pyogenes* isolates submitted in April to November to our clinical laboratory (which serves a population of approximately 2 million) and 13% of 125 invasive *S. pyogenes* isolates submitted in January to November within London to the national reference laboratory. This contrasted with *emm*1 strains, where a disproportionately high number accounted for invasive isolates (23%) compared with noninvasive isolates (11%) during the same time periods (unpublished data). At present, there are no experimental data that directly compare the invasiveness of the emergent clade with that of previous *emm*89 lineages. Using population-based mortality data, we found no evidence that the emergent *emm*89 clade, where associated with invasive infection, caused greater mortality than the original ST101 lineage in the United Kingdom (2).

The potential for certain lineages of *S. pyogenes* to undergo extensive recombination is of wider significance. It seems likely that future vaccines and/or diagnostic tests will target genes and gene products thought to be crucial for pathogenesis, and yet we have demonstrated that, at least in *emm*89, some factors can be lost or dramatically altered. The mechanisms explaining why and how these strains undergo recombination-related genome remodeling have not been determined but clearly require recognition and further evaluation.
